# 

**Published:** 1902-11

**Authors:** 


**This Journal and Atlanta Semi-weekly Journal one year for $1.00 cash in advance. No checks. Strictly to new subscribers.**

**This Journal and Atlanta Semi-weekly Journal one year for $1.00 cash in advance. No checks. Strictly to new subscribers.**

